# Tumor-related Microbiome in the Breast Microenvironment and Breast Cancer

**DOI:** 10.7150/jca.58986

**Published:** 2021-06-11

**Authors:** Na Wang, Tao Sun, Junnan Xu

**Affiliations:** 1 Department of Breast Medicine, Cancer Hospital of China Medical University, Liaoning Cancer Hospital, Shenyang, China, 110042.; 2 Department of Pharmacology, Cancer Hospital of China Medical University, Liaoning Cancer Hospital, Shenyang, China, 110042.; 3 Key Laboratory of Liaoning Breast Cancer Research, Shenyang, Liaoning, China.

**Keywords:** microbiome, breast cancer, gut microbiota, diversity

## Abstract

Despite the significant progress in diagnosis and treatment over the past years in the understanding of breast cancer pathophysiology, it remains one of the leading causes of mortality worldwide among females. Novel technologies are needed to improve better diagnostic and therapeutic approaches, and to better understand the role of tumor-environment microbiome players involved in the progression of this disease. The gut environment is enriched with over 100 trillion microorganisms, which participate in metabolic diseases, obesity, and inflammation, and influence the response to therapy. In addition to the direct metabolic effects of the gut microbiome, accumulating evidence has revealed that a microbiome also exists in the breast and in breast cancer tissue. This microbiome enriched in the breast environment and the tumor microenvironment may modulate effects potentially associated with carcinogenesis and therapeutic interventions in breast tissue, which to date have not been properly acknowledged. Herein, we review the most recent works associated with the population dynamics of breast microbes and explore the significance of the microbiome on diagnosis, tumor development, response to chemotherapy, endocrine therapy, and immunotherapy. To overcome the low reproducibility of evaluations of tumor-related microbiome, sequencing technical escalation and machine deep learning algorithms may be valid for standardization of assessment for breast-related microbiome and their applications as powerful biomarkers for prognosis and predictive response in the future.

## Introduction

Breast cancer is the most common cancer among females and is the leading cause of cancer-related deaths in females worldwide, with more than 600,000 deaths, accounting for 6.6% of global cancer-related deaths 1, 2. In the past few decades, growing evidence has confirmed that the gut microbiota plays an important role in carcinogenesis and response to anti-cancer therapy, such as in colorectal carcinoma, stomach tumors, and breast cancer 3. Researchers have gradually discovered that a great array of microbiota inhabit the normal breast and breast cancer tissue. A complex interaction occurs between the breast microbiota and breast carcinogenesis, and between the therapeutic response and drug resistance 4. Specific microbiota may be used as novel targets in breast cancer treatment and prevention. Regulation of the microbial community combined with treatment using individualized probiotics may decrease the risk of breast cancer and contribute to improving outcomes in patients, which will have a positive impact on the focus of breast cancer research.

## Microbiota in breast and its potential sources

**Figure 1:**
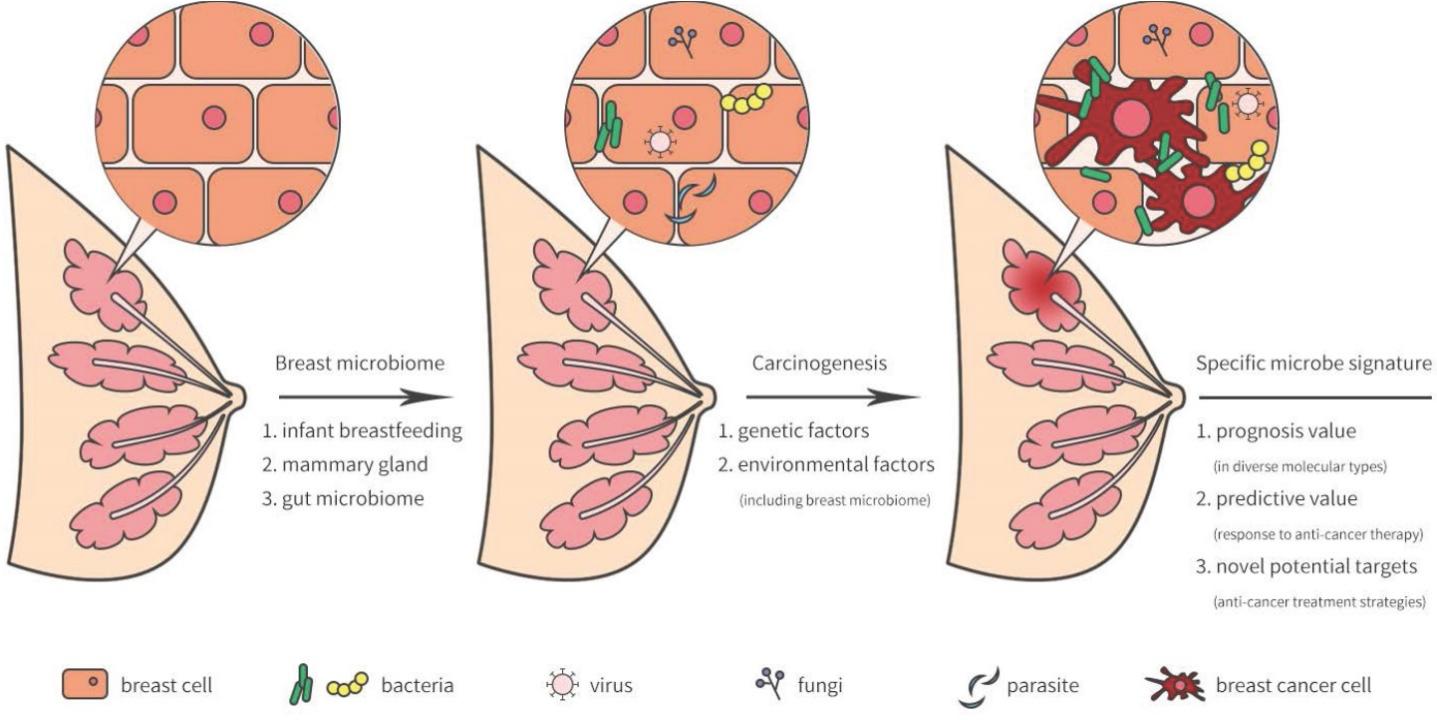
Breast-related microbiome in breast tissue and its potential carcinogenesis.

The breast was initially thought to be sterile, however, several recent studies have revealed that microorganisms reside in the breast tissue. Microbiota in the breast may actually originate from the skin, intestine, mammary gland, and breast milk during the early stages of life 5 (Figure 1). During breastfeeding, the infant skin and oral microbes may have had access to the mammary ducts and then bacteria may have persisted in the breast tissue. A landmark study revealed a previously unrecognized connection between mammary special bacteria and maternal gastrointestinal microbiota, subsequent activation of CD18+ cells and dendritic cells through an entero-mammary pathway. Additionally, CD18+ cells and dendritic cells have the capacity to transfer bacteria from the gastrointestinal lumen to the lactating mammary tissue 6. Chiba et al. 7 suggested that the mammary gland microbiota can also be modulated by diet and their findings indicated that the breast microbiome was characterized by plasticity and originated from gut microbiota.

Surgical procedures associated with bacterial infection may cause bacterial colonization in breast tissue. The major findings from the currently available literature indicates that infected tissue after breast reconstruction does indeed have a distinct microbiome, which derives from the microbiome located in the skin and at the opening of the milk ducts 8. Despite the activity of the host's immune system and antibiotic treatment, species commonly colonize the human skin or the smooth surfaces of biological materials to form a biofilm 9. As might be predicted, biopsies associated with biofilm communities were characterized by massive bacterial load, a pronounced inflammatory response, and clinical signs of more severe tissue involvement 10. Gram-negative bacteria are also involved in breast implant infections and this discrimination serves to determine the unique signature for prognosis. A study evaluating early infection of breast implants had found that the gram-negative organisms caused 27% of infections 11 and *Pseudomonas aeruginosa (P. aeruginosa)* was a common microorganism. The pyocyanin produced by *P. aeruginosa* can modulate the host immune response through multiple mechanisms to assist the escape from the host immune system and establish or exacerbate bacterial infection. Necrotizing soft tissue infection (NSTI) rarely appeared and mainly caused by a Gram-positive and Gram-negative bacteria. From the perspective of epidemiology, NSTI can be generally categorized into three main types. Type I is relatively rare and can also be caused by *P. aeruginosa* and is linked to polymicrobial etiology. A high prevalence of type II associated with large scale mortality rate of 18.7% continue to be reported. Type II is linked to *Streptococcus*, *Staphylococcus aureus* or *Staphylococcus epidermidis*, and is generally explosive. Clostridial expression belongs to Type III, which has a low incidence 12. These bacterial infections may have been related to bacterial location and the high mortality following NSTI in patients with breast implants or breast cancer-related surgery.

Breast is a favorable environment for location and growth of bacterial due to high percentage of adipose composition with lymphatic drainage and extensive vasculature. Studies have shown that Proteobacteria, Firmicutes, and Bacteroides in mammary are positively correlated with fatty acid metabolism by-products and fatty acid biosynthesis 13-15.

## Breast microbiota and breast carcinogenesis

As previously reported, lifestyle, age, race, and genetics served as high risk factors by facilitating tumor growth and increasing DNA damage that promote cancer progression 16-19. Indeed, breast environment have also been shown to increase cancer cell carcinogenesis and dissemination in breast cancer. A unique microbiome signature may induce carcinogenesis or the development of breast cancer. Meanwhile, the tumor microenvironment may provide a possible survival niche in which these microorganisms may continue to exist and evolve.

Compared with normal breast tissue, is the microbiota more strongly expressed in breast cancer tissue? And is there a significant difference in the composition of the microbial community? Xuan et al. 20 had characterized that *Sphingomonas* were enriched in normal breast tissue and *Methylobacterium* were enriched in breast cancer tissue. *Sphingomonas* highly presented in normal breast tissue and it may influence breast cancer progression in various ways, including estrogen metabolism and activation of Toll-like receptor (TLR) 5 -dependent pathways that inhibit development of breast cancer 21. Approximately, two thirds of estrogen receptor (ER)-positive breast cancer tissue is colonized by *Methylobacterium* 20. Microbial diversity is a critical indicator for evaluating community microbiome between breast cancer patients and healthy subjects 22. Nejman et al. 23 found that lower bacterial diversity presented in breast tumor samples than those in tumor-adjacent normal breast tissue or normal breast samples. Specific microbiome signatures and diversity may be a favorable biomarker for diagnosis and prognosis in patients with breast cancer.

Circumstantial evidence shows specific microbiota closely associated with the development of breast cancer, while taking medications with antibiotics or probiotics. Through a large-scale analysis of nearly 4 million women, Simin et al. 24 indicated that there was a specific and dose-dependent relationship between the use of antibiotics and breast cancer and classes of antibiotics varied somewhat in their association with breast cancer risk. Irregular use or overuse of antibiotics may increase risk of intestinal dysbiosis and reduces bacterial diversity 25, 26. Overuse of antibiotics is known to decrease lignan- enterolactone levels in the plasma, which have a direct influence on the microbiota and increase the risk for breast cancer 14, 22. Furthermore, antibiotics may reduce the amount of estrogens re-entering the enterohepatic circulation by influencing estrogen deconjugation, and thereby increase the fecal excretion of estrogens and decrease breast cancer risk 14. Prospective cohort studies have investigated the effects of probiotics on breast cancer. Animal experiments demonstrate a growing possibility that probiotics exert systemic anti-tumor effects via stimulating the immune system, regulating gut microbiota, and promoting the survival of healthy microflora 27, 28. Lakritz et al. 29 indicated that the probiotic *Lactobacillus reuteri* suppressed breast carcinogenesis and increased the sensitivity of mammary cells to apoptosis. Microbially-triggered antigen activated CD4+ CD25+ cells were proposed as a potential mechanism for such an effect. Oral administration of beneficial microorganisms such as *Bifidobacterium* and *Lactobacillus* in breast cancer patients can supplement and reactivate the immune system in cancer tissues, destroy tumor cells, and promote their elimination, and thus, exert positive effects on the prognosis of breast cancer 30, 31. Nevertheless, essential questions remain on the utility of antibiotics with specific classification and probiotics with specific strains and dosage in the clinical treatment of breast cancer. The improvement of microbiome diversity prepared for clinical application is a long process.

## Influences of specific viruses on breast cancer

It is now established that breast virome community may alter the development of breast cancer and influence clinical outcome. In recent decades, high risk *Human papillomavirus* (*HPV*), *Epstein Barr virus* (*EBV*), and *mouse mammary tumor virus* (*MMTV*) have been observed in breast cancer patients 32, 33.

Recently, studies on HPV in breast cancer have been reported, although they show conflicting results. Several molecular and epidemiological studies have suggested an involvement of HPV in breast carcinogenesis. An investigation by Salman et al. 34 reported that the HPV genome is detectable in breast cancer patients, and its high expression of onco-proteins E6 and E7 can disrupt the cell cycle by targeting p53 and Rb and thereby triggering malignant transformation and accelerating angiogenesis 32. Additionally, HPV interacted with cellular factors leads to breast carcinogenesis and modulate breast invasion 35, 36. Another study on breast cancer with viral positive had reported that HPV DNA were proposed in 16% of breast tumor tissues, and there is no association between viral transcription functionally and oncogenesis 37. This discrepancy may be explained by geographical location, race, or sample size of the reported studies, particularly geographic divergence 32. EBV, a gamma herpesvirus closely associated with epithelial malignancies, is widely known for its oncogenic properties. Farahmand et al. 38 showed that EBV leads to a 4.74-fold increase in risk of ductal breast cancer development. EBV encodes LMP1 and promotes cell proliferation and de-differentiation by NF-κB, MAPK, and phosphatidylinositol 3-kinase (PI3K)/AKT pathways 33, 38. However, Naushad et al. 33 indicated the profound effects of EBV on the development of breast cancer and its positivity in breast cancer patients was 24.4%, although no correlation was found between EBV positivity and stage, grade, or ER/ progesterone receptor (PR) status. MMTV, a retrovirus, has been extensively investigated as a cause of mammary tumors in mice, but its causative role in human breast cancer is far from being understood. Nartey et al. 39 identified the MMTV DNA sequence in 36% of human breast tumor samples and in 24% of non-cancerous breast tissues. However, unlike mouse cells, human cells do not have a cellular receptor for MMTV, and very little is known about how MMTV is able to enter human cells 40. The effects of viruses on the development of human breast cancer have not yet been fully elucidated. More research is needed to explore its potential molecular mechanisms and to discover effective cancer prevention and treatment strategies.

## Microbial signatures are associated with molecular subtypes of breast cancer

Breast cancer is a heterogeneous disease, the clinical classification sorts breast cancer into four major groups: Luminal A (ER+, [PR]-positive, human epidermal growth factor receptor 2 [HER2]-negative, low ki-67), Luminal B (ER+ and PR+/-, HER2+/-, or high ki-67), HER2-positive (HER2+ and ER-) and triple-negative (ER-/PR-/HER2-) based on immunohistochemical. These four subtypes represent biologically distinct disease entities, indicating that each breast cancer molecular type has a distinct response to chemotherapy, target therapy and immnotherapy. Current research has focused on the bacterial pathogens of breast cancer, together with fungi, viruses, and parasites. *Mobiluncus*, *Brevundimonas*, and *Actinomyces* were enriched in all subtypes. Fungal genera were also common detected in all types, including *Trichosporon*, *Rhodotorula*, *Geotrichum*,* Candida*, *Trichophyton*, and *Epidermophytonj*, however, more complex fungal diversity presented in ER+ breast cancer comparison to the triple-negative samples 4, 23, 41.

Are microbiological groups of different molecular types similar? Banerjee et al. 42 used a microarray-based approach called the “PathoChip” to determine the microbial signature for different breast cancer subtypes. The PathoChip is a microbial pathogen array containing probes, able to detect all publicly available virus sequences and hundreds of parasites, pathogenic bacteria and fungi 42, 43. The microbial signatures for each breast cancer molecular subtype are shown in Table 1 4, 41. The PathoChip detection results and case reports indicated that *Polyomaviridae*,* Plasmodium*, *Ascaris* and *Hepadnaviridae* were enriched in ER+/PR+ breast cancer 44, 45. Banerjee et al. 42 identified the unique microbial signatures linked with triple-negative breast cancer. *Polyoma viruses*, *herpesviruses*, *papilloma viruses*, *poxviruses*, *Arcanobacterium haemolyticum*,* Prevotella nigrescens*, *Pleistophora mulleris*,* Piedraia hortae*, *Trichuris trichura*,* Leishmania* in triple-negative breast tissues are much more frequently than normal tissues 42. In addition, plasmodium-derived molecules have been associated with antitumor properties in adult mice *in vivo* and *in vitro*, and may be used as targets for tumor immunotherapy in triple-negative breast cancer 46.

The qualitative and quantitative analysis of microbial signatures may provide beneficial diagnostic and prognostic information for patients with breast cancer, and will help provide clues for the design of novel treatment strategies.

## Therapeutic response by microbial in breast cancer

Breast tumors harbor their own specific microbiota varied on response to anti-cancer therapy. There is a giant confusion regarding the distinction between a predictive and prognosis biomarker. Microbiome biomarkers informs about a likely breast cancer outcome impartial of treatment received. Chiba et al. determined tumor tissues after neoadjuvant chemotherapy showed 65% increases in Pseudomonas (from 20% in baseline to 85%) via 16s rRNA sequencing that correlated with chemotherapy induce preferential growth or survival of these bacteria 7 Interestingly,* P. aeruginosa* at high concentrations inhibited the growth of the breast cancer cell lines MDA-MB-231, 4T1, 67NR and MCF7, while low concentrations showed opposite effects 7. These opposing effects may be regulated by secretions of *P. aeruginosa* metabolites. Pyocyanin, a toxin produced and secreted by *P. aeruginosa,* promotes cancer cell death and can also inhibit lymphocyte activity 7, 47. Studies have shown that *P. aeruginosa-*secreted factors enhance the ability of doxorubicin to inhibit tumor proliferation 48. Transcriptomic analysis of doxorubicin effects on *P. aeruginosa* indicate doxorubicin increased pqsH gene expression, which is a FAD-dependent monooxygenase required for the production of the Pseudomonas quinolone signal (PQS) 7. Chemotherapeutic efficacy can be modulated via PQS-mediated inhibition in two ways. One is inhibition of NF-kB by PQS may enhance doxorubicin-mediating anti-cancer activity. The other is PQS-mediated as a ferric iron chelator to reduce iron levels contributing to suppress breast tumor growth 49.

Selective estrogen receptor modulator (SERM) is mainly endocrine therapy for breast cancer, including tamoxifen and raloxifene, which also play an important role in preventing breast cancer. A sparking study indicated raloxifene can bind to *P. aeruginosa* PhzB2 to inhibit phenazine biosynthesis pathway to produce pyocyanin 50. Thus, raloxifene may be a suitable therapeutic drug for further investigation of *P. aeruginosa* infection. Additionally, Hussein et al. 51 reported that a polymyxin B and SERMs combined application also provides a novel therapy strategy for Gram-negative bacterial infections. Further investigation need to be explore the abundance of *P. aeruginosa ,* microbiome diversity and anti-tumor efficacy various endocrine therapy, such as SERM, selective estrogen receptor downregulator and aromatase inhibitor.

Bacteria targeting mitochondria can also have an impact on the resistance of host cells by influencing specific chemotherapeutic drugs 52, thereby affecting anti-tumor treatment response. Bacteria-induced mitochondrial DNA mutations, DNA damage response interference, mitochondrial function disorders, and changes in apoptosis regulation may lead to tumor cell growth and survival. Although it remains unclear how well the microbial mechanisms, and new therapeutic targets and predictive indicators to implement the known beneficial effects of microbiome on prognosis and prediction has still to be elucidated.

## Gut microbiome and drug metabolism in breast cancer

**Figure 2:**
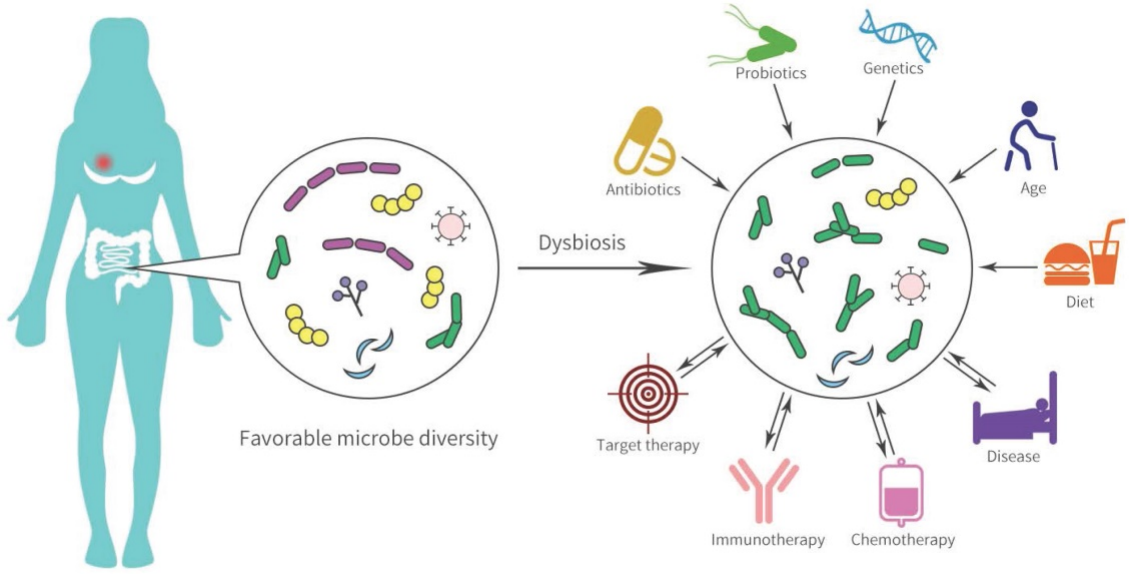
Gut microbial dysbiosis and disease pathogenesis in breast cancer.

The human body contains numerous bacterial and the gastrointestinal tract is the main organ enriched with microbiota. Many gut microbial-mediated mechanisms in tumor pathogenesis are complex and are considered underlying modifiable risk factors for breast cancer and response to therapy (Figure 2). In Goedert et al.'s study 53, the gut microbiota showed significantly lower diversity in 48 pretreatment postmenopausal breast cancer patients compared with 48 healthy controls. Ruminococcaceae, *Faecalibacterium*, and Clostridiaceae were enriched in patients with breast cancer, while levels of Lachnospiraceae and *Dorea* were low. The estrobolome present in the gut can accelerate the early deconjugation and interferes with the hydroxylation of estrogens, and thereby increases estrogen levels to generate an endogenous hormone environment. This endogenous hormone environment significantly increases the risk of hormone-dependent breast cancer cancer 54.

Studies have also investigated whether the gut microbiome differs according to ER/PR and HER2 status, obesity, and age of menarche. A unique bacterial composition and a less diverse microbiome was found among women with HER2+ breast cancers. Generally, the gut microbiota composition of patients with earlier age at menarche (≤ 11) and higher total body fat (≥ 46%) was characterized by reduced microbial diversity 3. Levels of Firmicutes of women associated with early menarche and HER2+ breast cancer were lowest (21.4%), intermediate among women who had later menarche and HER2+ (30.4%) or early menarche and HER2- (50.03%), and highest among those with later menarche and HER2+ (56.24%) 3. An unhealthy, inflamed gut causes breast cancer to become much more invasive and spread more quickly to other parts of the body. Gut microbial signatures may provide beneficial diagnostic and prognostic information for patients with breast cancer.

The gastrointestinal tract contains thousands of immunocytes and the gut microbiome accelerates the biotransformation of anti-cancer agents and the response to immunotherapy 55. Intestinal microorganisms catalyze many chemical reactions such as the removal of functional groups, the cracking of N-oxides, protein degradation, and opening of the thiazole ring, thereby accelerating the biotransformation of chemical drugs 56. The nitroreduction of the radiation sensitizer misonidazole and the hydrolysis of the anti-metabolite drugs methotrexate by gut microflora have been clarified 4, 56, 57. SN-38, an active metabolite of irinotecan, was converted to inactive SN38-G form by UDP-glucuronosyltransferases (UGTs) and caused severe diarrhea, which was correlated with polymorphism in UGT1A1 and the diversity of gut bacteria 55, 58. Diarrhea is frequently caused (75%) by lapatinib, an (epidermal growth factor receptor) EGFR/HER2 dual tyrosine kinase inhibitor for HER2+ advanced breast cancer. Recent evidence has shown that lapatinib-induced diarrhea might be involved in alterations to the gut microbiota. An increase in rats gut Proteobacteria, specifically Betaproteobacteria, was observed during a 14-day lapatinib treatment. After 28 days of treatment with lapatinib, the microbial diversity was markedly decreased, although, most diarrhea reactions occurred in the first 14 days after initiating treatment 59. High levels of Proteobacteria have been implicated in severe diarrhea or inflammatory diseases. However, microbial diversity has not been recognized as a potential driver of diarrhea, which is probably a consequence of persistent diarrhea. In preclinical models, elsiglutide was used for preventing severe lapatinib-associated diarrhea and microbial diversity of rats was reversely improved combining elsiglutide with lapatinib 59.

In addition to chemotherapy and targeted therapy, more encouraging investigations have indicated that specific microbiota may affect anti-cancer immunotherapy. Dysbiosis, prevalent in non-responders to anti-programmed death receptor (PD-1) therapy, may cause inflammation and the arrest of T cell differentiation into CD8^+^ effector cells, and has been associated with a significant reduction in the proportion of *Sphingomonas* 12, 60, 61. Oral Bifidobacterium can increase tumor cell control and contributes to interferon (IFN)-γ production by CD8+ tumor-specific T cells, and further increases the activation of intratumoral dendritic cells to improve anti-programmed death ligand (PD-L1) efficacy 60, 62. *Bacteroides fragilis* enhances anti-cytotoxic T lymphocyte associated protein (CTLA) 4 efficacy by activating Th1 cells and induces cross-reactivity to tumor neoantigens and bacterial antigens 60, 63. An *in vivo* mouse model established to study the anti-tumor effects showed that TLR4 agonists and CpG-oligodeoxynucleotide (CpG-ODN) suppressed tumor cell growth by overpowering immune regulators, tumor-infiltrating myeloid cells, and cytokine IL-10 leverls 64. Mice exposed to TLR4 ligand and lipopolysaccharide had a higher effectiveness in enhancing the anti-cancer response of immune cells than microbial-deficient and TLR4-deficient mice 64. Fluckiger et al. 65 demonstrated that the presence of enterococcal prophage engineered major histocompatibility complex class I-binding epitopes in intestinal microbiota correlated with long-term benefit of PD-1 blockade therapy. In 249 stool samples in patients with cancer, responders to PD-1 checkpoint inhibitors had higher levels of mucin Eckermann bacteria 66. The feces of breast cancer patients sensitive to PD-1 checkpoint inhibitors were transplanted into the intestinal tract of mice, and the mice showed good efficacy in PD-1 blockade 66. Thus, regulating the microbiome may become a new strategy to improve the efficacy for cancer immunotherapy.

Gut microbiota can influence the adverse effects and efficacy in patients with breast cancer by immune regulation and anti-cancer drug metabolism 67. Improving tumor-related gut microorganisms and its symbionts are considered a crucial approach in prevention and treatment of breast cancer in the future.

## Conclusion

Over the past few decades, tumor microbiota has attracted widespread attention in different fields, including breast cancer biology. Although, we are still a long way from fully understanding the specific and complex relationships between the microbiota and breast cancer. The study of the unique microbial characteristics in breast cancer, understanding its carcinogenesis, pathogenicity, or symbiosis in the tumor microenvironment will have a positive impact on future breast cancer prevention, early diagnosis, prognosis, and optimization of treatment strategies.

## Figures and Tables

**Table 1:** Distinct breast microbial features in diverse molecular subtypes of breast cancer

Cancer types	Bacterial signatures	Fungal signatures	Viral signatures	Parasitic signatures
ER+/PR+/HER2-	Arcanobacterium, Bifidobacterium, Cardiobacterium, Citrobacter, Escherichia	Filobasidilla, Mucor, Trichophyton	Hepadnaviridae	Brugia, Paragonimus, Ascaris, Plasmodium
ER+/PR+/HER2+	Bordetella, Campylobacter, Chlamydia, Chlamydophila, Legionella, Pasteurella	Penicillium	Birnaviridae, Hepeviridae Polyomaviridae Hepadnaviridae	Ancylostoma, Plasmodium Echinococcus, Schistosoma, Trichomonas, Trichostrongylus
ER-/PR-/HER2+	Streptococcus	Epidermophyton, Fonsecaea, Pseudallescheria	Nodaviridae	Balamuthia, Ascaris, Plasmodium
ER-/PR-/HER2-	Aerococcus, Arcobacter, Geobacillus, Orientia, Rothia	Alternaria, Malassezia, Piedraia, Rhizomucor		Leishmania, Contracaecum, Schistosoma, Necator, Trichuris Onchocerca, Toxocara, Trichinella

ER, estrogen receptor; PR, progesterone receptor; HER2, human epidermal growth factor receptor-2.

## Abbreviations

NSTI: necrotizing soft tissue infection
TLR: Toll-like receptor
ER: estrogen receptor
HPV: human papillomavirus
EBV: epstein barr virus
MMTV: mouse mammary tumor virus
PR: progesterone receptor
HER2: human epidermal growth factor receptor 2
PQS: pseudomonas quinolone signal
SERM: selective estrogen receptor modulator
UGTs: UDP-glucuronosyltransferases
EGFR: epidermal growth factor receptor
PD-1: programmed death receptor
PD-L1: programmed death ligand
CTLA-4: cytotoxic T lymphocyte associated antigen-4
